# The Anti-tumour Agent, Cisplatin, and its Clinically Ineffective Isomer, Transplatin, Produce Unique Gene Expression Profiles in Human Cells

**DOI:** 10.4137/cin.s802

**Published:** 2008-06-10

**Authors:** Anne M. Galea, Vincent Murray

**Affiliations:** School of Biochemistry and Molecular Genetics, University of New South Wales, Sydney, NSW 2052, Australia

**Keywords:** cisplatin, transplatin, gene expression, microarray

## Abstract

Cisplatin is a DNA-damaging anti-cancer agent that is widely used to treat a range of tumour types. Despite its clinical success, cisplatin treatment is still associated with a number of dose-limiting toxic side effects. The purpose of this study was to clarify the molecular events that are important in the anti-tumour activity of cisplatin, using gene expression profiling techniques. Currently, our incomplete understanding of this drug’s mechanism of action hinders the development of more efficient and less harmful cisplatin-based chemotherapeutics. In this study the effect of cisplatin on gene expression in human foreskin fibroblasts has been investigated using human 19K oligonucleotide microarrays. In addition its clinically inactive isomer, transplatin, was also tested. Dualfluor microarray experiments comparing treated and untreated cells were performed in quadruplicate. Cisplatin treatment was shown to significantly up- or down-regulate a consistent subset of genes. Many of these genes responded similarly to treatment with transplatin, the therapeutically inactive isomer of cisplatin. However, a smaller proportion of these transcripts underwent differential expression changes in response to the two isomers. Some of these genes may constitute part of the DNA damage response induced by cisplatin that is critical for its anti-tumour activity. Ultimately, the identification of gene expression responses unique to clinically active compounds, like cisplatin, could thus greatly benefit the design and development of improved chemotherapeutics.

## Introduction

The DNA-damaging agent, *cis*-diamminedichloroplatinum (II), cisplatin, is used extensively as a chemotherapeutic drug. In particular, it is successfully employed to treat ovarian and testicular carcinomas, as well as a range of other solid tumours (Adams et al. 1998; De Pree and Wils, 1989). However, dose-limiting toxic side effects and the occurrence of both acquired and intrinsic drug resistance in cells impose great limitations on cisplatin chemotherapy (Kelland, 2000; Siddik, 2003). While concerted efforts have been made towards developing cisplatin analogues with improved chemotherapeutic efficacy and reduced toxic side effects, only a few compounds have been clinically registered—the most notable of these being carboplatin and oxaliplatin (Hartmann and Lipp, 2003).

A major factor contributing to the current status of rationally designed analogues is a relatively poor understanding of the specific molecular events associated with cisplatin-induced tumour cell death. Although its detailed mechanism of action is presently unclear, it is generally thought that the covalent binding of cisplatin to cellular DNA and subsequent formation of bulky DNA adducts, mediate the cytotoxicity of this anti-cancer agent (Rosenberg, 1985; Wang and Lippard, 2005). Intra-strand DNA cross-links are the most common adducts formed, although inter-strand DNA cross-links and DNA-protein cross-links can also occur (Fichtinger-Schepman et al. 1985; Lippard and Hoeschele, 1979). The intra-strand DNA lesions preferentially form between the N-7 of adjacent guanine residues, inhibiting the passage of polymerases and thus interfering with DNA replication and RNA transcription inside target cells (Corda et al. 1992; Murray et al. 1992; Murray et al. 1998; Roberts and Thomson, 1979). While the general inhibition of RNA synthesis reduces the mRNA levels of many genes, an active cellular response to cisplatin damage can influence gene expression both positively and negatively and to varying extents, depending on the promoter (Evans and Gralla, 1992).

The active response to such drug-induced DNA damage consists of two key processes. These include the repair of DNA damage through the removal of cisplatin adducts and, where repair cannot be carried out successfully, the induction of cell death via apoptosis. While DNA repair and apoptotic pathways are considered to play the most significant role in determining cisplatin’s cytotoxic effect, various non-specific events associated with the drug-DNA interaction should also be taken into account. These non-specific events arise as consequences of cisplatin-DNA adduct formation and the overall inhibition of DNA and RNA synthesis. In addition, altered gene expression could also result from cisplatin-protein interactions (Perez, 1998) and the sequestration of transcription factors by cisplatin-DNA adducts. At present, little is known about the extent to which each of these active and non-specific responses contribute to cisplatin-induced cell death. This issue is addressed in the main aim of this work, which is to determine whether the regulation of specific genes in response to cisplatin treatment is crucial to the drug’s anti-tumour activity.

**Figure 1:**
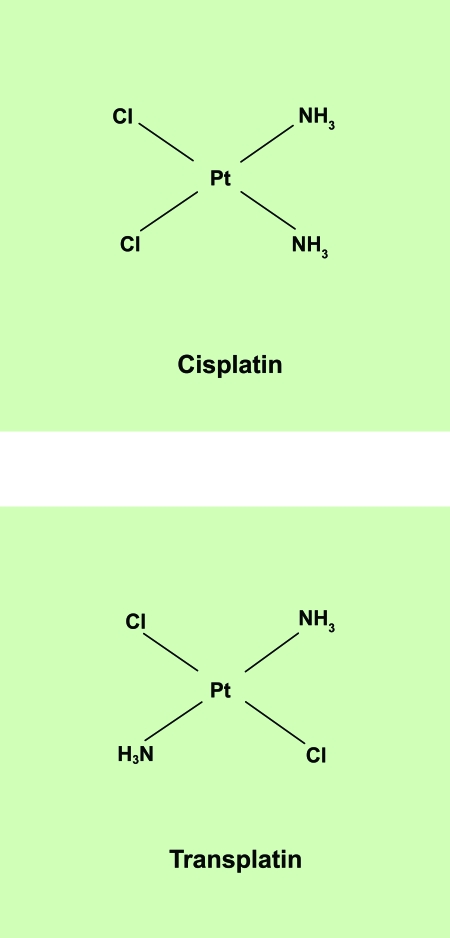
The chemical structures of cisplatin and transplatin The main focus of this study is the mechanism of action of the DNA-damaging anti-cancer drug, cisplatin. Although structurally similar, cisplatin’s isomer, transplatin, lacks the anti-tumour activity exhibited by

Oligonucleotide microarrays containing approximately nineteen thousand human genes were used to examine the broad effect of cisplatin on gene expression in human foreskin fibroblasts. The response of such a non-cancer cell line to DNA damage was considered to be particularly significant since most toxic side effects are due to the exposure of normal cells to the anti-tumour drug during chemotherapy. The gene expression profiles derived from these cells indicated that many genes consistently exhibited altered gene expression patterns due to cisplatin treatment. Rigorous methods for analysing the resulting microarray data were developed. Thorough normalisation techniques and a statistically robust procedure for estimating the significance of gene expression changes were implemented via the statistical computing environment, “R”. Using the software package, EASE, a method for exploring the biological themes among differentially expressed transcripts was also investigated. An attempt to clarify whether any of these molecular events are crucial to cisplatin’s anti-tumour activity was made by constructing similar expression profiles for the clinically inactive cisplatin isomer, transplatin (Fig. 1). Both compounds were found to consistently induce a common subset of gene responses in fibroblasts. However, thorough one- and two-sample statistical comparisons also indicated that several genes had significantly different expression levels in cisplatin-treated cells compared to transplatin-treated cells. The identification of gene expression responses unique to clinically active compounds, such as cisplatin, could have a range of implications on the design and development of improved chemotherapeutics.

## Materials and Methods

### Materials

Cisplatin, transplatin and Tri-Reagent were purchased from Sigma. Cell culture reagents and Superscript (II) Reverse Transcriptase were obtained from Invitrogen. Alamar Blue reagent was purchased through Astral Scientific. RNeasy columns were obtained from Qiagen. Cy3 and Cy5 mono-reactive fluorescent dyes were purchased from Amersham Biosciences. Human 19K oligonucleotide microarrays were produced by and purchased from The Clive and Vera Ramaciotti Centre for Gene Function Analysis, University of NSW.

### Cell Culture Conditions

The non-transformed human foreskin fibroblast cell line, FFbw002, was kindly donated by Noel Whitaker. Cells were grown in RPMI (Rosewell Park Memorial Institute) medium supplemented with 10% fetal bovine serum and maintained at 37 °C and 5% CO2 in a humidified incubator. Fibroblasts in log-phase growth were harvested prior to sub-culturing and drug treatment using mild trypsinisation.

### Cytotoxicity Assay

Cytotoxicity determinations for cisplatin and transplatin were performed using an Alamar Blue^™^ assay. FFbw002 Human foreskin fibroblasts were seeded at a density of 2 × 10^5^ cells/ml in 96-well, flat-bottomed microtitre plates in 100 μl of RPMI medium. Drugs (diluted in DMF) and controls were administered to cells in 100 μl of RPMI to give the final concentrations indicated: cisplatin (0.1mM to 100mM), transplatin (0.1 μM to 100 μM) and DMF (0.01% to 8% (v/v)). At least three replicates were performed for each administration. The plates were incubated at 37 °C in a humidified cell culture chamber with 5% CO_2_ for 5 hours. Twenty μl of Alamar Blue^™^ reagent was then added to each well and the plates were incubated for a further 3 hours. At this time, absorbance readings of the plates were immediately recorded using a Benchmark plate-reader with a sample wavelength of 570 nm and a reference of 595 nm. Following this eight-hour time point, plates were returned to the incubator and further absorbance readings were taken at 24 hours. Cell survival in the presence of drug was expressed as a percentage of cell growth in the drug-free DMF control corresponding to the% DMF in the drug treatment.

### Drug Treatment and Total RNA Extraction

Harvested fibroblasts were incubated at a concentration of approximately 5 × 10^6^ cells/ml RPMI medium, with varying concentrations of cisplatin or transplatin (each diluted in DMF), for 5 hours at 37 °C with 5% CO_2_. Total RNA was then isolated from cells using Tri-Reagent and the accompanying protocol for extracting total RNA from cultured cells (issued by Sigma). The integrity of total RNA was assessed using 1% agarose gel electrophoresis and its approximate concentration and purity estimated via UV spectrophotometry.

### cDNA Synthesis and Hybridisation

cDNA was synthesised using approximately 50 μg of total RNA in an oligo-dT(20mer)-primed reverse transcription reaction with Superscript II Reverse Transcriptase (Invitrogen). RNA was combined with 8 μl of 5× First Strand Superscript II Buffer (Invitrogen), 0.4nmol oligo-dT_20_, 10 μM DTT and made up to 32.2 μl with RNase-free H_2_O. Reactions were incubated for 5 minutes at 65 °C followed by 5 minutes at 42 °C. While at 42 °C, dATP, dGTP and dCTP nucleotides were added at a final concentration of 0.5 μM each, along with dTTP at 0.16 μM, aa(aminoalyl)-dUTP at 0.34 μM and 2 μl Superscript II Reverse Transcriptase. Reactions were then incubated for a further 2.5 hours at 42 °C. RNA was hydrolysed with the addition of 4ml of 50mM EDTA (pH8) and 2 μl of 10M NaOH to each sample, followed by an incubation of 20 minutes at 65 C. Reactions were neutralised with 4 μl of 5M acetic acid and then each sample was purified using a separate QIAquick PCR purification column (Qiagen). To couple mono-reactive fluorescent Cy-dyes (Amersham Biosciences) to the aminoalyl-dUTP moieties in target cDNA molecules, 9 μl of 0.1M NaHCO_3_ (pH9) and 2 μl of Cy5 (control samples) or Cy3 (drug-treated samples) were mixed with the appropriate samples. These reactions were left to incubate in a dark environment for 45 minutes at room temperature and then purified as above, using a separate QIAquick PCR purification column (Qiagen) for each sample. Hybridisation buffer was prepared with yeast tRNA (Sigma) at approximately 0.5 mg/ml and calf thymus DNA (Sigma) at approximately 0.5 mg/ml in DIG Easy Hyb (Roche). Concentrated Cy5(control) and Cy3(drug-treated) cDNA samples to be compared were combined directly with hybridisation buffer to a final volume of approximately 92 μl, heated for 5 minutes at 65 C and then allowed to cool to room temperature. Hybridisation mixtures were applied directly to microarray slides (Human 19K oligonucleotide arrays), each covered with a LifterSlip^™^ coverglass (ProSciTech) and then hybridised at 37 °C overnight (approximately 16 hours) in a humidified custom-made hybridisation chamber. LifterSlips^™^ were removed in 1 × SSC and slides washed three times in 1 × SSC/0.1% SDS at 50 °C. A final rinse in 1 × SSC was immediately followed by 10 minutes of centrifugation at 500 g to dry slides in preparation for scanning.

### Microarray Scanning, Data Acquisition and Processing

Microarrays were scanned using an Axon 4000A laser-based scanner and image data was acquired through associated GenePix Pro 3.0 software. Further data manipulation and statistical analyses (see below) were undertaken in the R statistical computing environment (www.r-project.org) with additional microarray-specific R-packages available from the BioConductor project (www.bioconductor.org). The R-function, *marrayGUI*, constructed by Mr Chris Bye (Westmead Millennium Institute) was used to input data into the R environment. Preliminary data processing involved the assignment of spot quality ‘weights’ to all data points. A “Robust Spline” method of intra-array normalisation (*limma* package in BioConductor), which utilises the spot quality weights determined above, was then applied to each slide. This approach accounts for both spatial- and intensity-dependent biases by fitting regression splines through data from individual print-tip groups and employing Empirical Bayes methods to shrink the print-tip curves towards a common value. Compared to standard “Print-tip Loess” normalisation procedures, such a technique is believed to introduce less “noise” into fairly good quality arrays that have little spatial variation. At this stage, unreliable data from spots with low quality weights due to low signal intensities or high spot background intensities was mostly filtered out so as to be excluded from further statistical analyses.

### Statistical Evaluation of Differential Gene Expression

Functions within the *limma* package in BioConductor were firstly employed to calculate a log-odds of differential expression (B-statistic) for each gene. This involved the fitting of gene-wise linear models through selected data and Empirical Bayes moderation of corresponding t-statistics. ‘One-’ and ‘Two-sample’ experimental design matrices were structured to allow the simultaneous assessment of differential expression within and between experimental conditions (microarray groups), respectively. The magnitudes of resulting B-statistics were then used to rank genes in order of evidence for differential expression. Transcripts considered to have a significant level of differential expression *within* a single test comparison (e.g. control vs treatment 1), were those highly ranked according to ‘one-sample’ B-statistic magnitudes and with average fold-changes greater than 1.4. Alternative procedures for multiple hypothesis testing available within the *multtest* package in BioConductor, were also investigated. In an ANOVA framework, F-statistics were calculated for each transcript using log expression ratio data from the three main test comparisons. As for the B-statistics, the magnitudes of these test-statistics were then used directly to identify transcripts most likely to be differentially expressed between the three test conditions.

### Gene Categories Over-represented Among Differentially Expressed Transcripts

Analysis tools implemented within the software package EASE (Expression Analysis Systematic Explorer) were used to annotate differentially expressed transcripts and explore the “biological themes” of significant gene subsets. EASE is freely available through the URL http://david.niaid.nih.gov/david/ease.htm.

## Results

The human gene expression response to cisplatin treatment was investigated using transcription profiling techniques. The use of oligonucleotide microarrays permitted the gene expression levels of many genes to be monitored simultaneously.

A human foreskin fibroblast cell line was employed and gene expression profiles were compiled for both cisplatin and its clinically ineffective isomer, transplatin. The transcriptional response of fibroblasts to drugs with different anti-tumour efficiencies could then be investigated.

### Alamar Blue^™^ cytotoxicity assays

**Figure 2:**
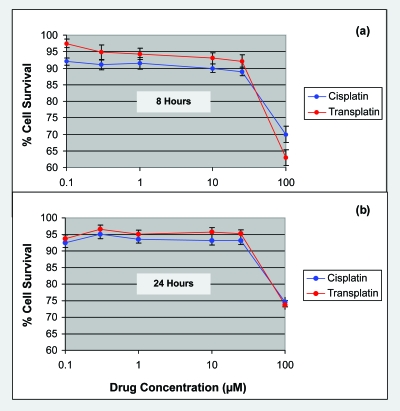
AlamarBlueTM Cytotoxicity Assay: Human fibroblasts treated with cisplatin and transplatin Graphs indicate the % Cell Survival at (**a**) 8 and (**b**) 24 hours for 0.1–100μM Cisplatin and Transplatin. The data points shown are for drug concentrations of 0.1, 0.3, 1.0, 10, 25 and 100 μM, and have been averaged across three replicate values. Error bars represent the standard error of the mean. (Note: Scale of x-axis is in logarithmic format).

Fibroblasts were firstly subjected to cytotoxicity assays with cisplatin and transplatin to determine optimal treatment conditions for inducing a clinically relevant gene expression response. Fibro-blasts were initially incubated at 37°C with drugs (or without drugs or controls) for 5 hours. At this time point, t = 5 hours, Alamar Blue^™^ reagent was added to each sample. Absorbance measurements were then taken at t = 8 and 24 hour time points and used to indicate the proportion of living cells remaining in each sample. Graphs in Figure 2 depict the percentage cell survival values determined for fibroblasts treated with 0.1–100 μM cisplatin or transplatin for 8 and 24 hours.

The cytotoxicity assay results for this particular fibroblast cell line indicated that there was a small to moderate difference between the cytotoxic effects elicited by cisplatin and transplatin. At the 8 hour time point, cisplatin was more toxic than transplatin at concentrations up to at least 25 μM. At 100 μM doses, transplatin appeared to induce slightly higher levels of cell death. For the 24 hour time point, however, cisplatin was consistently more cytotoxic to cells at all concentrations examined. Overall, drug concentrations ranging between 0.1 and 25 μM produced relatively low levels of cell death during both incubation periods. Within this dose range, the average cell survival estimates were 90% for cisplatin treatments and 95% for transplatin treatments. At drug concentrations above 25 μM, however, the cytotoxic effect of both compounds was more apparent.

In considering these results and those of the preliminary investigations, subsequent gene expression studies were conducted using 5 hour treatments with 1 μM cisplatin or transplatin. At this concentration, the cytotoxicity assay indicated that there was a slight difference between the toxic effects of the two isomers. To allow gene expression responses to *equitoxic* drug doses to be studied, a second transplatin concentration of 25 μM was also chosen for comparison. At this 8 hour dose, transplatin’s level of toxicity was approximately equivalent to that of 1 μM cisplatin. Similar differences in concentration have been required to achieve equitoxic cisplatin and transplatin doses in other studies (Burczynski et al. 2000; Sanchez-Perez et al. 1998; Sato et al. 1996). Furthermore, this concentration represented the lowest dose at which cytotoxicity levels were relatively low for both drugs.

### Microarray experiments, data acquisition and normalisation

All cell treatments were performed in quadruplicate. Total RNA was extracted from each cell population and used to synthesise fluorescently-labelled cDNA. cDNA samples derived from different treatments were then compared directly using human oligo-nucleotide microarrays containing approximately 19,000 human gene sequences and control elements. Microarray experiments, also carried out in quadruplicate, were performed for each of the following comparisons: DMF (control) vs 1 μM Cisplatin, DMF vs 1 μM Transplatin, DMF vs 25 μM Transplatin, 1 μM Transplatin vs 1 μM Cisplatin and 25 μM Transplatin vs 1 μM Cisplatin.

Following microarray scanning and data acquisition, raw results were processed and normalised as described in section 2.3.12. During these manipulations, the red and green fluorescence intensities were normalised relative to one another so that the green/red ratios approached an unbiased representation of relative expression levels. The ultimate aim of the normalisation process was to remove or reduce any systematic biases or errors in the results that were not due to the experimental condition(s) of interest (Smyth and Speed, 2003). In the current study, a “Robust Spline” method of intra-array normalisation was applied to data from each microarray using the *limma* (Linear Models for Microarray Data) package in BioConductor. The incorporation of spot quality weights determined in the preprocessing step (section 2.3.12(i)) controlled the extent to which each data point contributed to the normalisation calculations. This meant that unreliable data from spots with low quality weights due to low foreground or high background intensities were filtered out and excluded from the analysis. The “Robust Spline” function also accounted for both spatial- and intensity-dependent biases in the microarray data (Smyth and Speed, 2003; Yang et al. 2002).

**Figure 3:**
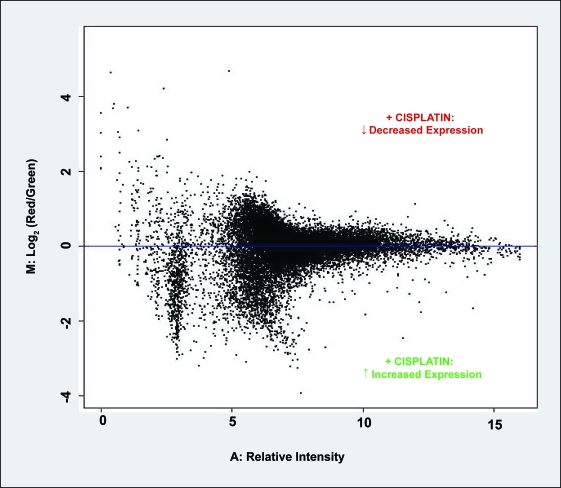
MA-plot of normalised data for DMF (control) versus 1 μM cisplatin treatment comparison M (Log_2_(expression ratio)) and A (relative intensity) values were averaged across 4 replicate microarrays. M values greater than zero indicate transcripts which responded to cisplatin with decreased expression levels, while M values less than zero indicate transcripts with increased expression levels after cisplatin treatment.

‘MA-plots’, as described by Yang et al., 2002, provided a convenient graphical means of representing the red (R) and green (G) fluorescence intensity data for each gene on the array. Within these plots, M_(y-axis)_ = log_2_(R/G) and A_(x-axis)_ = log_2_v(R × G). Thus, for each gene on the array, ‘M’ essentially describes the fluorescence intensity ratio, while ‘A’ represents the relative combined fluorescence intensity (Yang et al. 2002). Figure 3 shows a representative MA-plot for the normalised background-corrected data from DMF vs 1 μM Cisplatin treatment comparisons. Human gene transcripts with relatively up- or down-regulated expression levels due to cisplatin treatment are located below or above the ‘M = 0’ horizontal axis, respectively. In this study, MA-plots were particularly valuable in terms of identifying spot artifacts (or irregularities) and intensity-dependent trends in the log ratios (M-values). They were also employed for the purpose of monitoring and assessing the data normalisation procedures described above.

### Statistical significance evaluation – Overview

Having normalised the microarray data, the next step was to develop a statistically robust approach for defining differentially expressed transcripts. When examining such large sets of gene expression data, it is particularly crucial to address this issue since observed changes in expression may not always be a direct consequence of the test conditions. For example, altered expression patterns may result from variations in the data acquisition procedures or biologically insignificant fluctuations within the original cell populations that are not specifically related to the experimental conditions of interest. While many expression studies use a two-fold change in expression as the lower limit of significant gene expression changes, this approach can exclude valuable data and is not statistically flexible for use across multiple arrays. Instead, the current study adopted a statistically rigorous method for estimating and ranking the relative significance of observed gene expression changes.

Conducted within ‘R’, the main statistical procedure for defining differential expression implemented functions from the *limma* analysis package in BioConductor. These functions effectively facilitated the simultaneous analysis of comparisons between multiple RNA targets (that is, differentially-labeled cDNA populations). The key to this approach was a “design matrix” which was used to specify the desired treatments for comparison. This meant that data from multiple arrays could thus be examined simultaneously. The result was a log-odds of differential expression, or B-statistic, which was estimated for each gene in each treatment comparison. B-statistic magnitudes were then used to rank genes in order of evidence for differential expression.

Using the linear model approach described above, a “one-sample comparison” experimental design matrix was used to examine differential expression *within* separate microarray groups, where each ‘group’ represents one comparison performed on four replicate microarrays:

**Group 1.** DMF vs 1 μM cisplatin
**Group 2.** DMF vs 1 μM transplatin
**Group 3.** DMF vs 25 μM transplatin
**Group 4.** 1 μM transplatin vs 1 μM cisplatin
**Group 5.** 25 μM transplatin vs 1 μM cisplatin.

B-statistics were computed for all five comparisons. Genes with the highest B-statistics thus had the highest likelihood of being differentially expressed within each group. Next, data from the first three microarray groups was combined within a “two-sample comparison” design matrix. This technique was employed to assess the extent of differential expression *between* microarray groups, and thus between treatments. For example, the simultaneous analysis of Group 1 and Group 3 data revealed over 200 genes that were highly ranked for differential expression between the 1 μM cisplatin and 25 μM transplatin treatments. Following both one- and two-sample comparison B-statistic analyses, the ‘EASE’ software package was employed to search for over-represented gene categories amongst differentially expressed genes. This procedure allowed the biological themes of significant gene subsets to be investigated. In the subsequent sections of this chapter, the results of B-statistic analyses and over-representation studies are presented and discussed in further detail.

### One-sample comparison B-statistic analysis

One-sample comparison B-statistic analyses were successfully employed to estimate the relative significance of ‘apparent’ changes in gene expression levels between control and drug-treated samples. Transcripts with B-statistic values greater than zero were defined as exhibiting significantly different expression levels in response to treatment, while the highest B-statistics indicated genes with the highest likelihood of being differentially expressed. This study revealed many genes that were differentially expressed in each of the three treatments examined: 1 μM cisplatin, 1 μM and 25 μM transplatin (see Table 1). For example, in cisplatin-treated cells, 1227 transcripts were found to exhibit significantly higher expression levels, while 316 transcripts were classed with significantly *lower* expression levels. Table 2 describes the first 20 known genes that were significantly up- and down-regulated in response to 1 μM cisplatin. As might be expected, many of the differentially expressed transcripts were common to cisplatin and transplatin treatments, indicating some degree of consistency in the transcriptional response to both platinum isomers. Some of the transcriptional events common to all three treatments are also indicated in Table 2.

Using the expression analysis program, EASE, gene annotation tools were next employed to identify gene categories that were over-represented in each differentially expressed gene subset compared to what was represented on the microarrays. Individual gene categories were also classed into one of the following gene ontology (GO) systems: molecular function, biological process or cellular component. In each analysis, the gene category with the lowest ‘EASE score’ was considered to be the most ‘over-represented’ or significant category within that subset of differentially expressed transcripts. The first 20 most over-represented gene categories among transcripts up- and down-regulated by 1mM cisplatin are shown in Table 3. As indicated, most of these categories were also over-represented among the significant transcripts of transplatin treatments. It is thus possible that many of the genes associated with these molecular functions and biological processes are involved in a more general response to the toxic insult.

One-sample B-statistics were also calculated for the Group 4 and Group 5 microarray data: 1 μM transplatin vs 1 μM cisplatin and 25 μM transplatin vs 1 μM cisplatin. However, this analysis largely yielded negative B-statistic values, implying that there were no significant differences between the transcriptional responses to each treatment. Another possibility was that variations between the treatment-specific gene expression profiles were of a magnitude that could not be resolved using this technique, especially in the presence of relatively large systematic errors and inherent dye biases. While dye-swapping or reciprocal labeling techniques are often employed in attempts to counteract such biases, this approach is more amenable to microarray experiments in which the differences between cDNA populations are of a higher magnitude (for example, when comparing different cell lines). In the current study, the average ‘significant’ drug-induced gene expression changes were only approximately two-fold relative to control (non-treated) samples (see Table 1). Since a key aim of this work was to study treatment-specific gene expression events, an alternative method for comparing the transcriptional responses to each treatment was investigated. For this purpose, a *two-sample* comparison B-statistic analysis was employed to contrast the ‘control vs treatment’ profiles already established for cisplatin and transplatin.

### Two-sample comparison B-statistic analysis

**Figure 4:**
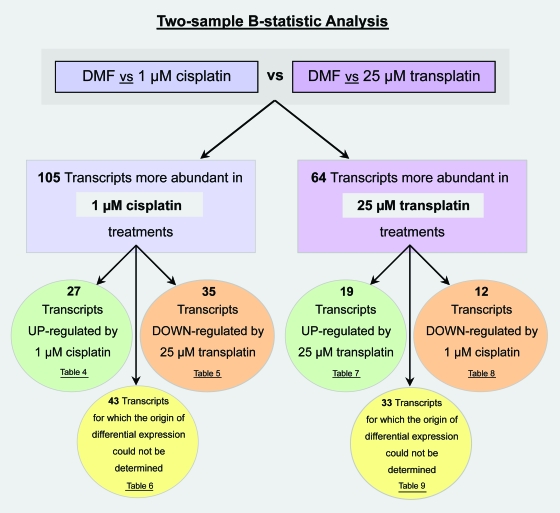
Overview of the two-sample comparison B-statistic analysis This approach facilitated the simultaneous comparison of data from ‘DMF (control) vs 1 μM cisplatin’ and ‘DMF (control) vs 25 μM transplatin’ microarray comparisons. Through the *inter*-array comparison of treatments labeled with the same dye, B-statistics were used to detect subtle differences in the expression responses to cisplatin and transplatin without the interference of dye biases. During this analysis, 105 transcripts were found to be significantly more abundant in the 1 μM cisplatin treatments while 64 transcripts were more abundant in the 25 μM transplatin treatments. The origin or cause of these differential expression events was then clarified by cross-comparing two-sample comparison results with those of the one-sample comparisons. Positive correlations then allowed significant transcripts to be classified as being actively up-regulated by cisplatin, down-regulated by transplatin, unregulated by transplatin or down-regulated by cisplatin. Other significant transcripts could not be classified into one of the four categories above, due to the absence of corresponding one-sample comparison data.

The two-sample B-statistic analysis facilitated the simultaneous comparison of microarray data from two different ‘control vs treatment’ experiments. Through the *inter*-array comparison of samples labeled with the same dye, B-statistics were used to detect more subtle differences in the expression responses to cisplatin and transplatin exposure, without the interference of dye biases. This approach, however, failed to recognise any consistently significant differences between the expression profiles induced by 5 hour 1 μM cisplatin and transplatin treatments. This implied a very high degree of similarity between the transcriptional responses of fibroblasts to these equivocal doses of cisplatin and transplatin. In contrast, two-sample comparison B-statistics readily detected significant differences between the responses to 1 μM cisplatin and 25 μM transplatin treatments. Altogether, 105 transcripts were found to be significantly more abundant in the 1 mM cisplatin treatments while 64 transcripts were more abundant in the 25 μM transplatin treatments (Table 1). For many of these genes, the origin or cause of their differential response could be clarified by cross-comparing the results of the two-sample comparisons with those of the one-sample comparisons. For example, transcripts that were more abundant in cisplatin treatments could be further classified as being ‘actively up-regulated by cisplatin’ if they were also present among the up-regulated genes of the one-sample ‘control vs 1 μM cisplatin’ comparisons. An overview of this classification process is presented in Figure 4. Of the 105 transcripts more abundant in cisplatin treatments, 27 were classified as being up-regulated by cisplatin (Table 4), 35 as being down-regulated by transplatin (Table 5), and 43 could not be classified into either group using the current one-sample data (Table 6). Of the 64 transcripts more abundant in transplatin treatments, 12 were classed as being up-regulated by transplatin (Table 7), 19 as being down-regulated by cisplatin (Table 8), and 33 did not correlate with any one-sample comparisons (Table 9).

Functions within EASE were also employed to detect gene categories that were significantly over-represented among the 169 transcripts differentially expressed between cisplatin and transplatin treatments. Tables 10 and 11 show some of the most over-represented categories among transcripts found to be more abundant in 1 μM cisplatin and 25 μM transplatin treatments, respectively.

## Discussion

The effect of the anti-tumour drug, cisplatin, on human gene expression was investigated using microarray-based transcription profiling techniques in human cells. The transcriptional response of human fibroblasts to a clinically relevant cisplatin dose was examined in detail using human 19K microarrays. Gene expression profiles were also compiled for transplatin, the therapeutically ineffective isomer of cisplatin. Statistically robust methods for assessing the significance of apparent changes in gene expression were then investigated. The first approach permitted the identification of human gene transcripts exhibiting significantly different expression levels in drug-treated compared to control samples. A second method was then used to reveal transcripts that were differentially expressed between the three drug treatments examined. During these comparisons, a subset of 169 transcripts was found to be differentially expressed between cisplatin and transplatin treatments. Gene ontology databases were used to recognise gene categories that were comparatively over-represented among the significant transcripts, compared to what was represented on the microarrays. This allowed the biological themes of the differential responses to cisplatin and transplatin treatments to be explored and further considered with respect to anti-tumour activity.

### Transcription profiling in drug-treated cells: overview

Toxic stress in cells can stimulate a range of biological responses, including the transcriptional modulation of genes regulating cell survival, DNA repair and cell death. It has thus been proposed that such complex patterns of induced gene expression changes could provide considerable insight into the mechanism of action of various toxic agents (Amen et al. 2002; Munson et al. 1999; Cabal et al. 2005; Hamden et al. 2002; Newton et al. 2004). For example, the discipline of toxicogenomics seeks to exploit the complexity of this response for the purpose of generating a molecular profile or signature that is characteristic of specific toxicant exposure (reviewed by (Gant and Zhang, 2005)). Furthermore, microarray-based genomic approaches can now serve as a powerful tool for exploring the molecular pathways and cellular processes that mediate the adverse responses to a particular compound (Caba et al. 2005).

The broad aim of the microarray experiments conducted in the present study was to investigate global gene expression responses to cisplatin exposure in human cells. The first significant outcome of this project was the establishment of distinct gene expression profiles for equitoxic doses of cisplatin and transplatin, relative to solvent (DMF) controls.

The second and somewhat more challenging aim of this study was to identify gene expression events that are unique to cisplatin treatment and could play an important role in its cytotoxic mechanism. For this purpose, transcription profiles constructed for cisplatin and its clinically ineffective isomer, transplatin, were compared. Many toxic compounds will lead to the induction of genes that are unrelated to anti-tumour activity. However, the use of active and inactive anti-tumour agents permits the identification of responses that contribute to anti-tumour activity and responses that do not contribute to anti-tumour activity. When sufficient data from different agents is combined, as in the case with cisplatin and transplatin, it may be possible to differentiate generic stress responses from compound-specific events (Burczynski et al. 2000; Gant and Zhang, 2005).

In order to extract accurate and informative data from this kind of gene expression study, there are several major experimental considerations that must also be addressed. One particular concern surrounds the biological system employed and its associated variables. In this project, non-transformed human foreskin fibroblasts were chosen as the initial cell type in which drug-induced gene expression profiles would be monitored. Most gene expression studies with cisplatin have been focused upon cancer cells against which cisplatin is successfully cytotoxic or ineffective due to problems of resistance (Clarke et al. 2004; Huerta et al. 2003; Lee et al. 2005; Macleod et al. 2005; Nakatani et al. 2005; Roberts et al. 2005; Takata et al. 2005; Toshimitsu et al. 2004; Wilson et al. 2005). Since both cancer and non-cancer cells of a patient are exposed to clinical treatments, a detailed look at the response of ‘normal’ cells to cisplatin may provide a more balanced insight into the toxic mechanism of this drug. Also, a number of important genes are “inactivated” in many tumour cells, such as p53 and retinoblastoma (Hill et al. 2005; Levine and Fleischli, 2000; Miller et al. 1996; Scheffner et al. 1991; Vaziri and Benchimol, 1999). Insight into the effect of cisplatin on the functional versions of such genes might thus be gained via the use of non-cancer cells in gene expression studies.

Biological variables that arise from experimental design, such as dose and time of exposure to the compound, also have a large impact on the analysis and subsequent interpretation of microarray data. These factors were thus given thorough consideration in the current study via several preliminary experiments. Filter macroarray studies revealed significant changes in gene expression upon the treatment of cells with 5 hour doses of cisplatin at concentrations between 1 and 50 μM. This study focused upon a moderate set of treatment conditions and a concentration of 1 μM cisplatin was chosen, since this dose falls within clinical ranges and has also been used in other microarray-based gene expression studies with cisplatin (Higuchi et al. 2003). Furthermore, this dose was not found to induce high levels of cell death in fibroblasts, according to cytotoxicity assay results. While gene expression profiles have already been used to define toxicity in various biological systems (Chang et al. 2003; Gant, 2002; van ‘t Veer et al. 2002), many of the chemical agents have been used at significantly toxic levels. It is now being recognised that more biologically relevant results are obtained under conditions of mild toxicity, particularly in the absence of any cellular or pathological changes (Gant and Zhang, 2005). Moreover, this approach has been successfully applied to identify differentially expressed genes during inflammatory responses to hexachlorobenzene (Ezendam et al. 2004). Together, these observations lend further support for the final choice of treatment conditions investigated here.

In order to compare the differential effects of cisplatin and transplatin treatments, two different concentrations of the clinically ineffective isomer were examined. In addition to an equimolar dose of 1 μM, a second approximately equitoxic dose of 25 μM transplatin was also selected for comparison with 1 μM cisplatin. Similar investigations have required transplatin at various doses between 2 and 100 times the concentration of cisplatin to achieve equitoxic effects (Burczynski et al. 2000; Sanchez-Perez et al. 1998; Sato et al. 1996). In addition, at least four-fold more transplatin than cisplatin adducts have been required to significantly inhibit transcription elongation in HeLa cells (Mello et al. 1995). However, in comparing 1 μM cisplatin and 25 μM transplatin doses, it is also possible that observed differences between gene expression profiles may arise purely due to the difference in drug ‘loads’. While the cytotoxicity assay indicated that relative toxicity levels were the same for these two treatments, the possibility of dose effects should also be taken into consideration. Interestingly, in the final microarray data, the relative magnitude of gene expression changes in 1 μM cisplatin-treated cells was generally more similar to the magnitude of changes in 25 μM transplatin treatments than in 1 μM transplatin treatments. This observation supports the final choice of isomer doses for comparison.

Apart from biological variables, another major experimental challenge relates to the intrinsic difficulties associated with the accurate measurement of gene expression – a problem that is further enlarged by the number of genes on a microarray. A means of overcoming such technical variations is through correct experimental design and the implementation of analytical procedures that ensure the data is as free from systematic errors as possible (Gant and Zhang, 2005; Zhang and Gant, 2004). In the current study, these issues were primarily addressed within the preliminary investigations. Microarray experiments using slides with smaller gene-sets were employed to trial hybridisation and array-scanning techniques with fluorescent dyes. Also, these trials used cDNA samples derived from HeLa and K562 cancer cells, since these cell lines were well characterised and known to be stable. Subsequent experiments conducted with larger arrays compared control (DMF) and cisplatin-treated samples in order to ascertain whether a stable response to the anti-tumour drug could be detected. As expected, the magnitude of the differences in gene expression levels between control and treated samples was significantly lower than that observed between the profiles of the two different cell lines. However, the fact that a subset of genes was found to exhibit significantly different expression levels in response to drug treatment satisfied a major goal of this project, which was to establish a gene expression profile for cisplatin-treated human cells using microarray technology.

The ultimate aim of this work then became to develop a complete system for reliably determining differentially expressed genes in drug-treated cells. In future research, such a test system could then be used with confidence to investigate the cytotoxic potential of other compounds (Burczynski et al. 2000; Caba et al. 2005; Gant and Zhang, 2005).

Oligonucleotide microarrays were used to investigate the transcriptional response of human fibroblasts to drug treatment. Through the use of refined normalisation procedures and rigorous statistical evaluation techniques, these experiments produced finite sets of genes that were classed as being differentially expressed in response to each of the three treatments examined. Additional cross-comparisons between different data sets led to the identification of a subset of genes that were differentially expressed between cisplatin and transplatin treatments.

The following sections briefly review and integrate the relevant literature relating to some of the more significant biological outcomes of the fibroblast microarray experiments. Particular emphasis is placed on the transcripts found to be differentially expressed between similarly toxic doses of cisplatin and transplatin. Since very rigorous and statistically robust methods for assessing differential gene expression were implemented in this part of the analysis, many of these transcripts are considered to be very good candidates for further investigation.

### Transcriptional responses common to cisplatin and transplatin treatments

The most important function of the one-sample B-statistic analyses was to identify genes with significantly different expression levels in control and drug-treated fibroblast samples. For all three treatments examined, results from the one-sample comparisons of microarray data clearly indicated that the cells exhibited a distinct transcriptional response to drug treatment (relative to control).

Differentially expressed transcripts were detected for each of the three treatments, forming separate transcription profiles for 1 μM cisplatin, 1 μM transplatin and 25 μM transplatin. Many of the differentially expressed transcripts were common to all three treatment profiles. However, considering the structural similarities between the two compounds and the relatively narrow dose range examined, this observation was not unexpected. A similar study by Burczynski and colleagues also describes a number of differentially expressed genes that are common to transcription profiles compiled for equitoxic doses of cisplatin and transplatin (Burczynski et al. 2000). Although it was not the purpose of this investigation to analyse these profiles in detail, it was interesting to note that some of the expression responses correlated well with observations from other DNA damage studies. For example, the interferon regulatory factors, IRF3 and IRF6, were both distinctly up-regulated in response to cisplatin and transplatin exposure in this study. The products of these genes are involved in a wide range of host defense mechanisms, and their activation by various environmental stresses, including DNA damage, has been well documented (Kim et al. 1999; Missiaglia et al. 2005). In the case of down-regulated transcripts, glutathione peroxidase 1 (GPX1) demonstrated significantly reduced expression levels in the cisplatin and transplatin treatments examined here. Similarly, decreased glutathione peroxidase expression and activity levels have already been observed following cisplatin exposure in a range of biological systems (Huang et al. 1997; Khynriam and Prasad, 2002; Naziroglu et al. 2004; Saad et al. 2004). Overall, such correlations clearly support the results obtained in this study.

EASE software was used to determine gene categories that were significantly over-represented among the transcripts of each treatment profile (see Table 3). Similarities between these expression profiles meant that many of the over-represented gene categories were also common to the three treatment groups. A number of the commonly over-represented categories corresponded to functions that have been previously implicated in the cellular response to toxic insult. Some of these involved genes with established roles in biological processes such as transport, cell growth and/or maintenance, signal transduction, cell proliferation and regulation of cell cycle. Other significant groups, such as lipid metabolism (which was the most over-represented category among up-regulated genes, see Table 3), have only few or no former associations with the cellular response to DNA damage (Vetoshkina and Dubskaia, 1993). Transcripts with roles in lipid metabolism included retinol dehydrogenase 16 (RODH-4), alkylglycerone phosphate synthase (AGPS) and apolipoprotein C-III (APOC3). Indeed, the complex series of events that results from drug-induced DNA damage involves multiple biological pathways, many of which are yet to be defined. Therefore, some of the significant genes identified here could prove to be novel regulators or mediators within the signal transduction pathways that are stimulated by DNA-adduct formation. Other transcripts may simply be part of the broader fibroblast response to toxic insult.

The next major goal of this investigation was to identify treatment-specific gene expression responses. While the microarray experiments that directly compared 1 μM cisplatin with 1 μM or 25 μM transplatin were originally designed to identify transcripts that were differentially expressed *between* treatments, one-sample B-statistic analyses failed to accurately reveal any such genes. It is most likely that systematic errors and biases in the microarray data significantly contributed to this outcome. However, as an alternative approach, two-sample B-statistic analyses were employed to facilitate the indirect comparison of expression profiles determined for the three ‘control vs treatment’ comparisons. Since these ‘treatment’ samples were all labelled with the same fluorescent dye (Cy3), this technique strongly reduced the likelihood of false positive results and dye-specific biases in the data. By implementing strict inclusion criteria and a robust definition for differential expression, the final outcome was a concise list of transcripts that were found to exhibit significantly different expression levels in cisplatin- and transplatin-treated fibroblasts.

### Gene expression profiles unique to cisplatin and transplatin treatments

The two-sample comparison approach for inter-array data analysis readily identified transcripts that were differentially expressed between cisplatin and transplatin treatments. Altogether, 105 transcripts were found to be significantly more abundant in cisplatin treatments (Tables 4–6), while 64 transcripts were expressed to a greater extent in transplatin treatments (Tables 7–9). The application of gene ontology (GO) mapping and pathway analysis to this data illustrated the way in which such gene expression events could be placed in the context of the underlying pathways and processes affected. Among the 169 significant transcripts, EASE revealed a number of distinct and sometimes opposing biological themes. A particularly interesting result was ‘negative regulation of cell proliferation’, which was one of the more dominant themes associated with cisplatin treatments (Table 10). Genes assigned to this category included IL6, IL1B, IL8, TGFB1, GAS1, and ETS1. Other over-represented gene categories that reflected cisplatin’s negative effects on cell growth were ‘apoptosis’, ‘programmed cell death’ and ‘cell cycle arrest’. Together, these themes are consistent with cisplatin’s effective cytotoxic mechanism.

In sharp contrast, EASE characterised transplatin’s differential transcript profile with a significant proportion of genes involved in the ‘positive regulation of cell proliferation’ (Table 11). These included DTR, CUL3 and IRS2. Other subsets of genes abundant in transplatin treatments had established roles in a range of more general nucleic acid metabolism and processing functions. Among these, RNA/mRNA binding and splicing properties were common, and ‘regulation of DNA-dependent transcription’ was represented by at least eight genes. Broadly, these themes suggest that cellular activities following transplatin exposure are focused at the level of DNA/RNA interactions and various transcriptional processes. Together with the ‘positive regulation of cell proliferation’ gene group, this could partly indicate the early effort or ability of cells to overcome the interference of transplatin adducts and maintain normal cellular processes.

Other aspects of transplatin’s differential transcript profile related to methods for circumventing the negative effects of platinum exposure. For example, several transcripts more abundant in transplatin treatments mapped to ‘heavy metal sensitivity/resistance’, ‘metal ion homeostasis’ and transport-related categories. This suggests that under the conditions employed here, some detoxification pathways may be more active in response to transplatin than to cisplatin. Some of these genes may play a role in processes that act to lower intracellular transplatin concentrations and thus help to prevent any drug-mediated interference with normal cell growth.

This interpretation would also be consistent with transplatin’s status as the therapeutically inactive isomer.

In contrast, many of the dominating biological processes and molecular functions associated with cisplatin’s differential expression profile were directly concerned with the immediate fate of a cell (Table 10). ‘Regulation of cell proliferation’, ‘cell cycle arrest’ and ‘apoptosis’ are prime examples of such crucial processes. Interestingly, these categories were not as significantly represented among the transcripts of transplatin’s unique profile. However, since both adduct recognition and repair processes can differ significantly for damage induced by cisplatin and transplatin, this observation may reflect the early differential response of cells to the two isomers (Ciccarelli et al. 1985; Hansson and Wood, 1989; Heiger-Bernays et al. 1990; Huang et al. 1994; Jamieson and Lippard, 1999; McA’Nulty and Lippard, 1996; Mello et al. 1995; Zamble et al. 1996). Also, while cisplatin and transplatin have been shown to inhibit DNA synthesis in a similar manner (Bernges and Holler, 1988; Ciccarelli et al. 1985; Harder et al. 1976; Heiger-Bernays et al. 1990; Mello et al. 1995; Salles et al. 1983), their differential effects on RNA transcription are also widely acknowledged (Brabec and Leng, 1993; Corda et al. 1993; Evans and Gralla, 1992; Mello et al. 1995; Mymryk et al. 1995; Zlatanova et al. 1998). Thus, it seems likely that transcript profiles would also reflect such differences.

Overall, the results described here suggest that, even at low doses, cisplatin may elicit a more complex stress response in cells compared to transplatin. This might then imply that the need to determine the immediate fate of a cell is more urgent in response to cisplatin exposure. Supporting this notion was the abundance of transcripts pertaining to the immune system, communication and cell signalling processes in cisplatin treatments. In an attempt to further characterise the molecular events that may be associated with the anti-tumour activity of cisplatin, some of the genes found to be specifically regulated by cisplatin (Tables 4 and 8), were considered in more detail.

Inter-array two-sample B-statistic comparisons effectively revealed at least 169 transcripts that were differentially expressed between cisplatin and transplatin treatments. The incorporation of results from the one-sample ‘control vs treatment’ comparisons provided a means to clarify the origins of a subset of these differential responses. These subsets, presented in Tables 4, 5, 7 and 8, describe the more consistent and statistically significant transcriptional responses that were differentially elicited by cisplatin and transplatin in this study. A brief functional review of some of the better characterised genes that were specifically up- (Table 4) or down-regulated (Table 8) by cisplatin gave insight into some of the processes that may contribute towards the anti-tumour activity of this compound.

### Genes specifically up-regulated in response to cisplatin

The most common biological themes among the transcripts up-regulated by cisplatin involved cytokines, the regulation of cell proliferation, and other aspects of the cellular immune/defense response. Classed within each of these categories were the cytokines IL-1B, IL-6 and IL-8, which were all consistently up-regulated in response to cisplatin treatments. These molecules are important mediators of the inflammatory response and are also involved in a diverse range of cellular activities such as cell proliferation, differentiation, angiogenesis and apoptosis. Their ability to exert direct cytotoxic effects on tumour cells (Gaffney and Tsai, 1986; Poppenborg et al. 1999) or potentiate the effects of certain anti-tumour agents, has also been demonstrated (Benchekroun et al. 1993). Therefore, the collective increase in cytokine transcripts observed in the cisplatin-specific expression profile was considered to be significant. Several studies have already demonstrated a significant rise in interleukin-1 levels after cisplatin treatments in cultured cells (Shi et al. 1998; Sodhi and Pai, 1992; Suresh and Sodhi, 1991; Toubi et al. 2003) and in patients undergoing chemotherapy (Baiocchi et al. 1993). Furthermore, other groups have specifically reported that IL-1 enhances the sensitivity of tumour cells to cisplatin and that synergistic interactions between IL-1 and cisplatin may actually enhance p53-dependent apoptosis (Benchekroun et al. 1993; Poppenborg et al. 1999; Song et al. 1998).

The behaviour of cytokines IL-6 and IL-8 has also been examined extensively, particularly in cancer patients (Baiocchi et al. 1993; Bhalla et al. 2000; Darai et al. 2003). As for IL-1, an increased production of IL-6 and IL-8 in response to cisplatin has also been observed (Baiocchi et al. 1993; Shi et al. 1998; Toubi et al. 2003). In this study, the cisplatin-induced increase in IL-1B, IL-6 and IL-8 expression is thus consistent with previous findings and with the role of these cytokines as key biochemical modulators in a range of important biological functions.

Other elements of cisplatin’s unique transcript profile relate to important signalling events that can affect DNA synthesis and cellular proliferation. For example, NMB, ITPR3 and PRKCBP1 are all implicated in the phosphoinositide cascade, which involves the activation of protein kinase C (PKC) and subsequent PKC-mediated effects. In this study, these three genes were specifically up-regulated in response to cisplatin exposure (Table 4). Neuromedin B (NMB) is a bombesin-like peptide found chiefly in the central nervous system and gastrointestinal tract (Minamino et al. 1983; Minamino et al. 1985; Namba et al. 1985). This peptide demonstrates autocrine and paracrine growth factor activity in some carcinomas (Lach et al. 1995; Moody et al. 1992; Mukai et al. 1987; Otsuki et al. 1987), but in its role as a bifunctional regulator of cell growth, it can also significantly inhibit cell growth when at high levels (Dobrzanski et al. 1993). ITPR3 and PRKCBP1 have other roles in the PKC transduction pathway. ITPR3 is a second messenger receptor that acts as an intra-cellular calcium channel (Maranto, 1994), while PRKCBP1 (protein kinase C binding protein 1) functions as an anchor for activated protein kinase C isoenzymes (Fossey et al. 2000). At present, the precise role of the PKC transduction pathway in the cellular response to cisplatin is yet to be fully clarified (Grunicke et al. 2003; Hayakawa et al. 1999). However, the cisplatin-enhanced expression of NMB, ITPR3 and PRKCBP1 in the current study may shed light on some of the key factors involved. Moreover, the absence of these expression events in the response to transplatin damage suggests a possible role for the phosphoinositide cascade in cisplatin’s cytotoxic mechanism.

At least three transcripts among those specifically up-regulated by cisplatin had established or tentative growth suppressing properties: GAS1 (growth arrest specific 1), CDX1 (caudal type homeobox transcription factor 1) and AIM1 (absent in melanoma 1). GAS1 is an integral plasma membrane protein directly involved in the negative regulation of cell proliferation and, in some cases, apoptosis (Del Sal et al. 1995; Evdokiou and Cowled, 1998; Zamorano et al. 2004). CDX1 encodes an intestine-specific transcription factor that demonstrates both pro-oncogenic functions and growth-inhibitory effects (Domon-Dell et al. 2003; Lynch et al. 2003). AIM1, a novel non-lens member of the betagamma-crystallin superfamily, is a putative suppressor of human malignant melanoma and is associated with the control and experimental reversal of tumourigenicity (Ray et al. 1997). To date, GAS1, CDX1 and AIM1 have not been implicated in the response of cells to cisplatin damage.

In contrast, cisplatin-induced transcripts that have been shown to influence cell growth in a positive manner include CDC25B, G0S2 and AGER. CDC25B (cell division cycle 25B) and G0S2 (putative lymphocyte G0/G1 switch gene) both exert their most prominent effects through the regulation of the cell cycle. CDC25B and other CDC25 genes encode protein threonine/tyrosine phosphatases that drive cell cycle progression through the activation of cyclin dependent kinases. With obvious growth-promoting properties, CDC25B over-expression has been demonstrated in a number of cancers including head, neck, gastric, ovarian, esophageal and prostate tumours, as well as non Hodgkin’s lymphoma (Broggini et al. 2000; Gasparotto et al. 1997; Hernandez et al. 1998; Kishi et al. 2002; Kudo et al. 1997; Miyata et al. 2000; Ngan et al. 2003). While the precise function of G0S2 is yet to be established, its major role in cell cycle regulation is believed to involve the switch of lymphocytes from G0 to G1 phases (Cristillo et al. 1997; Russell and Forsdyke, 1991). AGER (advanced glycosylation end product-specific receptor), or RAGE, is generally a tumour-associated antigen and has been shown to stimulate cell proliferation and survival (Adams et al. 2002; Arumugam et al. 2004; Eichmuller et al. 2002; Hsieh et al. 2003). Also, at least one study has implicated AGER in the up-regulation of the pro-inflammatory cytokine IL-6 (Dukic-Stefanovic et al. 2003), which was also induced in the current study.

### Genes specifically down-regulated in response to cisplatin

Within this study, many of the transcripts found to be specifically down-regulated in response to cisplatin (Table 8) appeared to be more consistently associated with tumour-promoting or growth-stimulating effects. This finding is significant because the relative inhibition of such effects via decreased gene expression could contribute to the efficient anti-tumour mechanism of cisplatin. Among the down-regulated transcripts, genes that have been specifically linked with the potential for promoting growth and/or proliferation include: ephrin-B2 (EFNB2), angiopoietin-like 4 (ANG-PTL4), diphtheria toxin receptor (DTR) and splicing factor proline/glutamine rich (SFPQ). Such associations are outlined briefly, below.

The ephrin-B2 gene encodes a member of the ephrin (EPH) family which, along with the EPH-related receptors, comprise a large subfamily of receptor protein-tyrosine kinases (Bennett et al. 1995). Members have been most strongly been implicated in mediating developmental events, particularly in the central nervous system (Takasu et al. 2002) and in erythropoiesis (Suenobu et al. 2002). EFNB2 expression has also been associated with cellular proliferation (Batlle et al. 2002; Steinle et al. 2003), cell migration (Steinle et al. 2003), angiogenesis (Noren et al. 2004) and the progression of a wide range of human cancers, including malignant melanoma (Vogt et al. 1998), small cell lung carcinoma (Tang et al. 1999), osteosarcoma (Varelias et al. 2002), endometrial cancer (Takai et al. 2001), colon/colorectal carcinoma (Liu et al. 2004) and breast cancer (Noren et al. 2004). In fact, the capacity of the EFNB2 ligand to increase the potential for growth, tumourigenicity and metastasis in many of these tumour cells is becoming increasingly apparent (Noren et al. 2004; Takai et al. 2001; Vogt et al. 1998).

The ANGPTL4 gene encodes an angiopoietin-like secreted glycoprotein (Kim et al. 2000; Yoon et al. 2000). It is one of the targets of the nuclear receptor, peroxisome proliferator-activated receptor gamma (PPARgamma), and has proposed roles in adipose differentiation, lipid metabolism, glucose homeostasis and angiogenesis (Chen et al. 2004; Kersten et al. 2000; Le Jan et al. 2003; Lee and Evans, 2002; Schmuth et al. 2004; Yoon et al. 2000; Zhu et al. 2002). Like EFNB2, ANGPTL4 has also been associated with the development of a range of cancers, including colorectal cancer, renal cell carcinoma, bladder tumours and gastric cancer (Kaneda et al. 2002; Landi et al. 2003; Le Jan et al. 2003; Yoshimura et al. 2003).

DTR, also known as the heparin-binding EGF-like growth factor (HB-EGF), encodes a transmembrane protein that interacts with membrane protein DRAP27/CD9 to form the functional diphtheria toxin receptor (Fen et al. 1993; Hayes et al. 1987; Higashiyama et al. 1995; Iwamoto et al. 1994). In keeping with the growth-promoting characteristics of other down-regulated transcripts in this study, DTR demonstrates growth factor activity and mitogenic activity (Higashiyama et al. 1991; Higashiyama et al. 1995). The capacity of the diphtheria toxin receptor to stimulate cell migration and proliferation has also been documented (Higashiyama et al. 1991; Iivanainen et al. 2003; Kiso et al. 2003), as has its contribution to the tumourigenesis of gastric epithelial cell cancers (Murayama et al. 2002; Wallasch et al. 2002).

SFPQ (splicing factor proline/glutamine rich) is a novel and essential pre-mRNA splicing factor (Patton et al. 1993). It has been implicated in both early and late steps of pre-mRNA splicing and is required in spliceosome formation (Patton et al. 1993). With a number of roles in various nuclear processes, SFPQ has also been linked with virally-mediated steroidogenesis and oncogenesis (Song et al. 2004). Another positive association with cellular proliferation is the DNA-pairing activity exhibited by this protein, which has been directly implicated in the reestablishment of stalled replication forks (Akhmedov and Lopez, 2000).

Among the remaining transcripts in this study with reduced expression levels in response to cisplatin exposure, the metallothionein-1L (MT1L) and transferrin receptor (TFRC) genes have been most thoroughly studied and characterised. Although not directly associated with the promotion of cell growth, MT1L and TFRC respectively play major roles in metal detoxification and iron metabolism, both of which are essential cellular processes. Furthermore, the relative effect of cisplatin treatment on MT1L and TFRC has already been addressed in a number of studies, as discussed below.

Metallothioneins are intracellular metal-binding proteins that are generally found to confer drug resistance when induced in tumour tissues. Metallothionein-mediated cisplatin resistance, for instance, is well documented (Bakka et al. 1981; Basu and Lazo, 1990; Endresen and Rugstad, 1987; Kelland et al. 1992; Satoh et al. 1994; Suganuma et al. 2003; Yang et al. 1994). Although cisplatin has also been found to induce metallothionein expression in tumour cells (Bauman et al. 1991; Farnworth et al. 1989; Harford and Sarkar, 1989; Kondo et al. 2003; Matsumoto et al. 1997; Singh and Koropatnick, 1988; Zhang et al. 1995), induction appears to be dependent on the protein isoform and the drug-resistant status of the cells (Kinsler and Bell, 1985; Nakano et al. 2003). Furthermore, metallothionein has also been shown to exhibit a biphasic transcriptional response to DNA damage in which early expression levels are largely depressed (Hansen et al. 1997). In considering these observations, it is thus possible that reduced metallothionein expression could contribute to early drug-induced anti-proliferative effects by lessening the chemo-protection that is usually afforded by increased metallothionein levels. In further support of the findings reported in this thesis, comparative studies with cisplatin and transplatin have shown that the inactive isomer interacts at a significantly faster rate with metallothionein, and does not appear to induce its biosynthesis (Farnworth et al. 1989; Harford and Sarkar, 1989; Zelazowski et al. 1984; Zhang et al. 1995).

As introduced above, the transferrin receptor gene, TFRC, encodes a glycoprotein with an essential role in iron metabolism (Omary and Trowbridge, 1981; Schneider et al. 1984). Several interesting relationships between cisplatin and TFRC have also been revealed. Firstly, the ability of cisplatin to bind transferrin is well established, and there is evidence that the cisplatin-transferrin complex can be transported into cells via the transferrin receptor (Allardyce et al. 2002; Gullo et al. 1980; Hamada 1988; Head et al. 1997; Sykes et al. 1985). In another study, cisplatin-induced transferrin modulation was found to be accompanied by severe spermatogenic damage in rat testes (Nambu and Kumamoto, 1995). This observation may be significant because it has the potential to provide insight into the enhanced sensitivity of testicular carcinomas to cisplatin-based therapies.

Various studies have also documented the reduced expression of TFRC mRNA after cisplatin treatment (Marazzi et al. 1991; Parodi et al. 1988; Tonini et al. 1986), an observation that is consistent with the findings reported in the current study. Moreover, Marazzi and colleagues (1991) observed that this effect correlated well with low TFRC protein expression and the inhibition of DNA synthesis and cellular proliferation. Conversely, a number of studies have reported a distinct correlation between higher levels of TFRC expression and increased cellular proliferation in cancer cells (Miyamoto, 1992; Moura et al. 2004; Staber et al. 2004). Together, these findings suggest a more defined role for TFRC in promoting cellular growth and proliferation – again, a common theme among the genes down-modulated by cisplatin in this study. Overall, the connection between TFRC and cisplatin damage is potentially an interesting one, and certainly worthy of further study.

## Conclusion

In conclusion, the current study has utilised microarrays to identify a number of genes that are differentially expressed in human cells in response to cisplatin and transplatin treatments. However, the functional interpretation of transcriptional events revealed by microarray analysis still presents a major challenge. Researching the known properties and functions of such genes is indeed a small but important step towards understanding their biological relevance in the experimental context of interest. In the present study, such investigations provided insight into several gene expression responses that are uniquely elicited by cisplatin with respect to its clinically ineffective isomer, transplatin. This data has indicted a number of genes that would be strong candidates for further gene function analysis that can mimic the effect of cisplatin at the gene expression level.

## Figures and Tables

**Table 1:** The number of differentially expressed transcripts identified within each key treatment comparison For each control vs treatment comparison, significant responses are divided into up- and down-regulated gene subsets. For two-sample indirect treatment comparisons, differentially expressed transcripts were identified as being either more abundant in 1μM cisplatin treatments or 25μM transplatin treatments. The average and highest expression fold-changes within each significant subset are also indicated.

	Treatment comparison	Significant gene subset	Number of significant genes (B 3 0)	Average fold-change	Highest Fold-change (gene name)
One-sample	Control (DMF) vs 1μM Cisplatin	Transcripts up-regulated by 1μM Cisplatin	↑ 1227	2.81	**19.27** (REM protein, REM)
		Transcripts down-regulated by 1μM cisplatin	↓ 316	2.02	**3.63** (cDNA DKFZp564D2071)
	Control (DMF) vs 1μM Transplatin	Transcripts up-regulated by 1μM transplatin	↑ 1293	2.78	**16.51** (REM protein, REM)
		Transcripts down-regulated by 1μM transplatin	↓ 352	1.95	**3.28** (MHC class II DPw3-alpha-1 chain)
	Control (DMF) vs 25μM Transplatin	Transcripts up-regulated by25μM transplatin	↑ 1319	2.83	**20.37** (REM protein, REM)
		Transcripts down-regulated by25μM transplatin	↓ 561	1.91	**3.63** (cDNA DKFZp564D2071)
Two-sample	Control (DMF) vs 1μM Cisplatin and 25μM Transplatin	Transcripts more abundant in1μM cisplatin	105	1.71	**3.29** (interleukin 1-beta (IL1B)
		Transcripts more abundant in 25μM transplatin	64	1.65	**2.36** (cDNA FLJ12109 fis)

**Table 2:** One-Sample Comparison B-statistic Analysis: The first 20 known genes with significantly (a) higher and (b) lower expression levels in cells treated with 1 μM cisplatin Transcripts are ranked in order of evidence for differential expression, according to one-sample comparison B-statistics and corresponding M and P-values. For each transcript with at least two data points, the average fold-change was calculated from the replicate values shown. Overall, 1245 transcripts were found to be significantly up-regulated in response to 1 μM cisplatin, while 352 were significantly down-regulated following treatment. Transcripts highlighted in yellow demonstrated the same expression response in transplatin treated cells.

Accession #	Full gene name	Average fold-Change	B-statistic	M	P-value
**(a)** Transcripts Up-regulated in response to 1 μM Cisplatin					
U12767	Human mitogen induced nuclear orphan receptor (MINOR) mRNA	**8.27**	**8.243**	−3.040	0.00026
M26747	Human c-erbA mRNA, complete cds	**9.70**	**8.081**	−3.265	0.00026
X59845	Human mRNA for a Krab domain upstream to Kox 8 gene	**5.50**	**8.045**	−2.503	0.00026
D17259	Human HepG2 3′-region MboI mRNA, clone hmd5d07m3	**3.18**	**8.025**	−1.666	0.00026
AF223389	PCGEM1 gene, non-coding mRNA	**7.44**	**7.965**	−2.888	0.00026
NM_006515	SET domain and mariner transposase fusion gene (SETMAR) mRNA	**7.18**	**7.716**	2.848	0.00026
NM_006666	RuvB (E coli homolog)-like 2 (RUVBL2) mRNA	**5.53**	**7.690**	−2.504	0.00026
NM_003708	microsomal NAD+-dependent retinol dehydrogenase 4 (RODH-4) MRNA	**6.84**	**7.488**	−2.764	0.00026
NM_004010	dystrophin (muscular dystrophy, Duchenne and Becker types), (DMD), transcript variant Dp427p2, mRNA	**4.83**	**7.354**	−2.270	0.00028
NM_003743	nuclear receptor coactivator 1 (NCOA1), mRNA	**6.23**	**7.326**	−2.630	0.00028
NM_005677	single strand of homotrimeric collagen-like tail subunit of asymmetric acetylcholinesterase (COLQ) mRNA	**3.87**	**7.235**	1.951	0.00029
NM_005558	ladinin 1 (LAD1) mRNA	**5.01**	**7.161**	−2.322	0.00029
NM_016063	CGI-130 protein (LOC51020), mRNA	**4.58**	**7.142**	−2.194	0.00029
NM_001571	interferon regulatory factor 3 (IRF3) mRNA	**5.61**	**7.112**	−2.405	0.00030
AF298152	mRNA polymerase III transcription initiation factor B″ short mRNA	**4.46**	**7.047**	−2.155	0.00030
L48516	paraoxonase 3 (PON3) mRNA, 3′-end of cds	**4.85**	**6.840**	−2.273	0.00036
AF274863	Secretory pathway component Sec31B-1 mRNA, alternatively Spliced	**2.47**	**6.754**	−1.301	0.00039
AF205632	zinc finger protein 106 (ZFP106) mRNA, complete cds	**5.75**	**6.750**	−2.510	0.00039
NM_012332	Mitochondrial Acyl-CoA Thioesterase (MT-ACT48), mRNA	**4.17**	**6.667**	−2.058	0.00039
NM_004375	COX11 (yeast) homolog, cytochrome c oxidase assembly protein, MRNA	**3.24**	**6.591**	−1.685	0.00041
**(b)** Transcripts Down-regulated in Response to 1 μM Cisplatin					
NM_002450	metallothionein 1L (MT1L) mRNA	2.20	5.218	1.133	0.00063
NM_003539	H4 histone family, member B (H4FB) mRNA	3.56	4.002	1.758	0.00100
NM_002307	lectin, galactoside-binding, soluble, 7 (galectin 7) (LGALS7) mRNA	2.31	3.707	1.206	0.00108
M87941	Human carcinoma cell-derived Alu RNA transcript, clone ALU20	2.48	3.468	1.301	0.00122
NM_015885	PCF11p homolog (LOC51585), mRNA	1.91	3.435	0.925	0.00110
AF040247	erythroid differentiation-related factor 1 mRNA, partial cds	3.07	3.379	1.573	0.00130
M92273	Human dihydropyridine-sensitive calcium channel HFCC, 3′-end intron 4	2.30	3.272	1.200	0.00133
NM_005066	splicing factor proline/glutamine rich (polypyrimidine tract-binding protein-associated) (SFPQ) mRNA	1.70	3.191	0.768	0.00123
U62823	Human small nuclear RNA U6atac, partial sequence	1.77	3.029	0.826	0.00133
Y10206	H.sapiens mRNA for CD64 protein	3.37	2.943	1.791	0.00158
NM_012348	olfactory receptor 89 (OLFR89), mRNA	2.28	2.902	1.178	0.00161
X96644	H.sapiens mRNA for U45a small nuclear RNA	2.12	2.791	1.083	0.00166
AY010111	cadherin-23 (CDH23) mRNA, partial cds	2.17	2.513	1.113	0.00187
U50539	Human BRCA2 region, mRNA sequence GT605	2.21	2.470	1.138	0.00192
NM_005553	keratin, cuticle, ultrahigh sulphur 1 (KRN1), mRNA HERV-H homolog/ya88a12s.1 homolog (A_T-rich region, putative retrotransposon)	2.20	2.423	1.132	0.00195
S83307	[human, phytohemaglutinin-stimulated peripheral T-cells, mRNA ]	2.11	2.389	1.091	0.00205
Y16701	mRNA from HIV associated non-Hodgkin’s lymphoma (clone hl1–72)	2.06	2.387	1.039	0.00199
AF124819	T84 colon carcinoma cell IL-1beta regulated HSCC1 mRNA	2.29	2.374	1.166	0.00205
NM_014387	linker for activation of T cells (LAT), mRNA	1.78	2.323	0.829	0.00190
NM_005110	Glutamine-fructose-6-phosphate transaminase 2 (GFPT2) mRNA	1.70	2.252	0.767	0.00188

**Table 3:** One-Sample Comparison B-statistic Analysis: The first 20 most significantly over-represented gene categories among transcripts with significantly (a) higher and (b) lower expression levels in cells treated with 1 μM cisplatin The number of transcripts in each gene category (List Hits) is also presented as a percentage of all the transcripts belonging to that category that were actually present on the microarray (Population Hits). Gene categories denoted with an asterisk were also significantly over-represented among transcripts demonstrating the same expression response in transplatin treated cells.

Rank	GO system	Gene category	List hits	Population hits	% Population hits	EASE score
**(a)** Gene categories over-represented among **up-regulated** transcripts						
1	Biological Process	lipid metabolism *	**100**	474	21	1.79 × 10^−19^
2	Biological Process	transport *	**210**	1646	13	8.23 × 10^−13^
3	Biological Process	membrane lipid metabolism *	**27**	93	29	1.05 × 10^−08^
4	Biological Process	phospholipid metabolism *	**21**	59	36	1.44 × 10^−08^
5	Biological Process	lipid biosynthesis *	**36**	164	22	6.93 × 10^−08^
6	Molecular Function	transporter activity *	**178**	1542	12	1.29 × 10^−07^
7	Molecular Function	porter activity *	**37**	184	20	3.77 × 10^−07^
8	Molecular Function	electrochemical potential-driven transporter activity *	**37**	185	20	4.33 × 10^−07^
9	Biological Process	phospholipid biosynthesis *	**14**	33	42	6.86 × 10^−07^
10	Molecular Function	dynein ATPase activity *	**9**	14	64	3.15 × 10^−06^
11	Molecular Function	carrier activity *	**60**	406	15	4.40 × 10^−06^
12	Biological Process	lipid transport *	**16**	54	30	1.50 × 10^−05^
13	Cellular Component	intermediate filament cytoskeleton *	**18**	69	26	1.81 × 10^−05^
14	Biological Process	membrane lipid biosynthesis *	**14**	43	33	2.05 × 10^−05^
15	Molecular Function	motor activity *	**24**	112	21	2.10 × 10^−05^
16	Molecular Function	microtubule motor activity *	**14**	44	32	2.51 × 10^−05^
17	Molecular Function	lipid transporter activity *	**16**	58	28	3.52 × 10^−05^
18	Biological Process	alcohol metabolism *	**35**	211	17	0.00007
19	Biological Process	endocytosis *	**22**	107	21	0.00010
20	Biological Process	steroid metabolism *	**22**	109	20	0.00014
**(b)** Gene categories over-represented among **down-regulated** transcripts						
1	Molecular Function	signal transducer activity *	**32**	1901	1.7	0.0006
2	Molecular Function	sugar binding *	**6**	99	6.1	0.0021
3	Molecular Function	receptor activity *	**21**	1163	1.8	0.0037
4	Biological Process	cell surface receptor linked signal transduction *	**19**	968	2.0	0.0047
5	Molecular Function	transmembrane receptor activity *	**14**	756	1.9	0.0195
6	Biological Process	response to abiotic stimulus *	**11**	494	2.2	0.0202
7	Biological Process	development *	**25**	1646	1.5	0.0215
8	Biological Process	cell communication *	**37**	2744	1.3	0.0216
9	Cellular Component	membrane *	**48**	4039	1.2	0.0321
10	Biological Process	neuropeptide signaling pathway *	**4**	87	4.6	0.0512
11	Biological Process	perception of abiotic stimulus *	**7**	280	2.5	0.0527
12	Biological Process	sensory perception *	**7**	292	2.4	0.0621
13	Biological Process	cell-cell adhesion *	**5**	166	3.0	0.0757
14	Biological Process	perception of external stimulus	**7**	308	2.3	0.0761
15	Biological Process	neurogenesis *	**8**	392	2.0	0.0831
16	Biological Process	endocytosis *	**4**	107	3.7	0.0838
17	Biological Process	signal transduction *	**28**	2167	1.3	0.0839
18	Biological Process	G-protein coupled receptor protein signaling pathway *	**10**	564	1.8	0.0923
19	Biological Process	perception of chemical substance	**3**	54	5.6	0.0949
20	Cellular Component	nucleoplasm	**12**	787	1.5	0.1132

**Table 4:** Two-sample comparison B-statistic analysis: transcripts significantly up-regulated in 1 μM cisplatin treatments (relative to both DMF (control) and 25 μM transplatin treatments) Transcripts are ranked in order of evidence for differential expression according to one-sample comparison B-statistics and corresponding P-values.

Accession #	Gene symbol	Full gene name	FOLD-change	B-statistic	P-value	Representative gene category	EASE score
M15330	IL1B	interleukin 1, beta	**3.29**	7.88	0.0002	negative regulation of cell proliferation	0.0023
M21551	NMB	neuromedin B	**2.43**	7.83	0.0002	cell communication	0.0279
AK022077	BAALC	brain and acute leukemia, cytoplasmic	**2.55**	4.96	0.0040		
NM_015714	G0S2	putative lymphocyte G0/G1 switch gene	**2.75**	4.72	0.0047	regulation of cell cycle	0.0001
AK027167	FLJ23514	hypothetical protein FLJ23514	**1.79**	4.41	0.0055	protein metabolism	0.8321
AB036432	AGER	advanced glycosylation end product-specific receptor	**1.86**	4.14	0.0058	response to pest/pathogen/parasite	0.0021
NM_005512	GARP	glycoprotein A repetitions predominant	**2.79**	3.75	0.0077	response to external stimulus	0.0075
M14584	IL6	interleukin 6 (interferon, beta 2)	**1.70**	3.53	0.0089	positive regulation of cell proliferation	0.1881
NM_002224	ITPR3	inositol 1,4,5-triphosphate receptor, type 3	**1.88**	3.12	0.0120	signal transducer activity	0.2901
AL157504		unknown	**2.20**	2.68	0.0167		
AK021624	C5orf13	chromosome 5 open reading frame 13	**2.13**	2.62	0.0170		
AK026341	FLJ22688	hypothetical protein FLJ22688	**2.25**	2.54	0.0180	cell growth and/or maintenance	0.2968
AK024330	PAPPA	pregnancy-associated plasma protein A	**1.71**	2.29	0.0206	zinc ion binding	0.0667
NM_002048	GAS1	growth arrest-specific 1	**2.01**	2.25	0.0206	negative regulation of cell proliferation	0.0023
NM_014400	C4.4A	GPI-anchored metastasis-associated protein homolog	**1.67**	2.03	0.0217	cell communication	0.0279
NM_018006	FLJ10140	hypothetical protein FLJ10140	**1.87**	1.82	0.0252	tRNA metabolism	0.5373
NM_019885	P450RAI-2	cytochrome P450 retinoid metabolizing protein	**1.70**	1.58	0.0290	oxidoreductase activity	0.8690
NM_004358	CDC25B	cell division cycle 25B	**1.76**	1.57	0.0290	regulation of cell cycle	0.0001
AK022207	MLPH	melanophilin	**1.67**	1.33	0.0333		
U83115	AIM1	absent in melanoma1	**1.69**	1.29	0.0339	cell adhesion	0.3235
NM_015524	C6orf4	chromosome 6 open reading frame 4	**1.54**	1.03	0.0370	cell communication	0.0279
NM_000022	ADA	adenosine deaminase	**1.42**	0.93	0.0382	response to pest/pathogen/parasite	0.0021
AF085920		unknown	**1.53**	0.60	0.0460		
M17017	IL8	interleukin 8	**1.64**	0.56	0.0460	negative regulation of cell proliferation	0.0023
NM_006332	IFI30	interferon, gamma-inducible protein 30	**1.56**	0.52	0.0469	immune response	0.0038
NM_001804	CDX1	caudal type homeo box transcription factor 1	**1.66**	0.28	0.0564	morphogenesis	0.0427
AF233453	PRKCBP1	protein kinase C binding protein 1	**1.62**	0.01	0.0664	transcription\, DNA-dependent	0.9772

**Table 5:** Two-sample comparison B-statistic analysis: transcripts significantly down-regulated in 25 μM transplatin treatments (relative to both DMF (control) and 1 μM cisplatin treatments) Transcripts are ranked in order of evidence for differential expression according to one-sample comparison B-statistics and corresponding P-values.

Accession #	Gene symbol	Full gene name	FOLD-change	B-statistic	P-value	Representative gene category	EASE score
NM_007286	SYNPO	synaptopodin	**2.13**	4.50	0.0055	hydrolase activity	0.7902
NM_003028	SHB	SHB (Src homology 2 domain containing) adaptor protein B	**1.87**	4.40	0.0055	cell communication	0.0279
NM_005438	FOSL1	FOS-like antigen 1	**2.06**	4.33	0.0056	positive regulation of cell proliferation	0.1881
AL359568		unknown	**1.86**	4.25	0.0057		
NM_005940	MMP11	matrix metalloproteinase 11 (stromelysin 3)	**1.86**	4.14	0.0058	zinc ion binding	0.0667
NM_000362	TIMP3	tissue inhibitor of metalloproteinase 3 (Sorsby fundus dystrophy, pseudoinflammatory)	**2.10**	3.63	0.0082	response to external stimulus	0.0075
NM_015719	COL5A3	collagen, type V, alpha 3	**1.94**	3.36	0.0102	cell adhesion	0.3235
AF075060		unknown	**1.56**	3.35	0.0102		
NM_018579	MSCP	mitochondrial solute carrier protein	**1.65**	3.27	0.0109	cell growth and/or maintenance	0.2968
AK024488	FLJ21438	hypothetical protein FLJ21438	**1.79**	2.96	0.0139		
NM_002997	SDC1	syndecan 1	**1.63**	2.51	0.0182	cytoskeletal protein binding	0.5656
NM_003040	SLC4A2	solute carrier family 4, anion exchanger, member 2 (erythrocyte membrane protein band 3-like 1)	**1.58**	2.48	0.0185	cell growth and/or maintenance	0.2968
M16006	SERPINE1	serine (or cysteine) proteinase inhibitor, clade E (nexin, plasminogen activator inhibitor type 1), member 1	**1.82**	2.16	0.0208	enzyme regulator activity	0.1463
U79458		unknown	**1.52**	2.14	0.0208		
U90908	RhoGAP2	Rho GTPase activating protein 2	**1.51**	2.07	0.0214	tRNA metabolism	0.5373
AB033050	RAI17	retinoic acid induced 17	**1.74**	2.04	0.0216	zinc ion binding	0.0667
AK001058		unknown	**1.54**	1.52	0.0292		
AF086287		unknown	**1.69**	1.49	0.0292		
NM_007182	RASSF1	Ras association (RalGDS/AF-6) domain family 1	**1.42**	1.39	0.0318	cell communication	0.0279
NM_006288	THY1	Thy-1 cell surface antigen	**1.62**	1.27	0.0339	cell growth and/or maintenance	0.2968
AK026108	SEMA4B	sema domain, immunoglobulin domain (Ig), transmembrane domain (TM) and short cytoplasmic domain, (semaphorin) 4B	**1.64**	1.27	0.0339	neurogenesis	0.7846
NM_002103	GYS1	glycogen synthase 1 (muscle)	**1.56**	1.20	0.0353	energy derivation by oxidation of organic compounds	0.5979
AL390147	FAM20C	family with sequence similarity 20, member C	**1.52**	1.20	0.0353		
NM_003565	ULK1	unc-51-like kinase 1 (C. elegans)	**1.48**	1.13	0.0353	kinase activity	0.5026
AF238083	SPHK1	sphingosine kinase 1	**1.50**	1.01	0.0370	regulation of cell cycle	0.0001
NM_006497	HIC1	hypermethylated in cancer 1	**1.59**	0.91	0.0383	negative regulation of cell cycle	0.0876
NM_014623	MEA	male-enhanced antigen	**1.57**	0.87	0.0395	gonad development	0.1233
NM_015923	SLC7A5	solute carrier family 7 (cationic amino acid transporter, y+ system), member 5	**1.38**	0.85	0.0396	cell growth and/or maintenance	0.2968
AB007871	SRGAP2	SLIT-ROBO Rho GTPase activating protein 2	**1.4**	0.68	0.0432	cell communication	0.0279
NM_003289	TPM2	tropomyosin 2 (beta)	**1.42**	0.67	0.0432	cell motility	0.0688
NM_005178	BCL3	B-cell CLL/lymphoma 3	**1.45**	0.47	0.0486	regulation of cell cycle	0.0001
NM_005730	CTDSP2	CTD (carboxy-terminal domain, RNA polymerase II, polypeptide A) small phosphatase 2	**1.72**	0.41	0.0507	oncogenesis	0.3266
NM_006373	VAT1	vesicle amine transport protein 1 homolog (T californica)	**1.59**	0.35	0.0530	zinc ion binding	0.0667
NM_017458	MVP	major vault protein	**1.52**	0.03	0.0635	cell cycle	0.0025
AK025665	C22orf20	chromosome 22 open reading frame 20	**1.70**	0.03	0.0635	integral to membrane	0.6857

**Table 6:** Two-sample comparison B-statistic analysis: transcripts abundant in 1 μM cisplatin treatments (relative to 25 μM transplatin treatments) for which a one-sample origin of differential expression could not be determined.

Accession #	Gene symbol	Full gene name	FOLD-change	B-statistic	P-value	Representative gene category	EASE score
AF086149		unknown	1.88	4.40	0.0055		
AL137705		unknown	1.98	3.90	0.0072		
NM_007066	PKIG	protein kinase (cAMP-dependent, catalytic) inhibitor gamma	2.04	3.80	0.0075	phosphate metabolism	0.7057
NM_000861	HRH1	histamine receptor H1	1.67	2.84	0.0153	response to pest/pathogen/parasite	0.0021
AL353944		unknown	1.61	2.74	0.0167		
AK025758		unknown	1.60	2.72	0.0167		
NM_002205	ITGA5	integrin, alpha 5 (fibronectin receptor, alpha polypeptide)	1.92	2.70	0.0167	cell adhesion	0.3235
AK024429	CLG	likely ortholog of mouse common-site lymphoma/leukemia GEF	1.64	2.42	0.0194	response to external stimulus	0.0075
NM_005718	ARPC4	actin related protein 2/3 complex, subunit 4, 20kDa	1.53	2.19	0.0208	cell motility	0.0688
AB028949	KIAA1026	KIAA1026 protein	1.72	2.17	0.0208	morphogenesis	0.0427
NM_003946	NOL3	nucleolar protein 3 (apoptosis repress sor with CARD domain)	1.76	2.15	0.0208	apoptosis	0.2968
NM_014631	SH3MD1	SH3 multiple domains 1	1.56	1.70	0.0275	cell communication	0.0279
J03077	PSAP	prosaposin (variant Gaucher disease and variant meta chromatic leukodys trophy)	1.83	1.69	0.0275	sphingolipid metabolism	0.2202
AK026383		unknown	1.54	1.59	0.0290		
NM_012098	ANGPTL2	angiopoietin-like 2	1.71	1.57	0.0290	development	0.2095
NM_000623	BDKRB2	bradykinin receptor B2	1.66	1.15	0.0353	response to pest/pathogen/parasite	0.0021
AB028998	TENC1	tensin like C1 domain-containing phosphatase	2.00	1.15	0.0353	cell communication	0.0279
NM_005127	CLECSF2	C-type (calcium dependent, carbohydrate-recognition domain) lectin, superfamily member 2 (activation-induced)	1.53	1.14	0.0353	response to pest/pathogen/parasite	0.0021
NM_001878	CRABP2	cellular retinoic acid binding protein 2	1.47	1.12	0.0353	cell communication	0.0279
NM_017720	STAP2	signal-transducing adaptor protein-2	1.50	1.11	0.0353	Cell communication	0.0279
NM_001397	ECE1	endothelin converting enzyme 1	1.46	1.00	0.0377	Cell communication	0.0279
NM_014096		unknown	1.63	0.96	0.0381		
NM_004604	STX4A	syntaxin 4A (placental)	1.48	0.87	0.0395	cell growth and/or maintenance	0.2968
AF217967	PP1057	hypothetical protein PP1057	1.69	0.78	0.0429		
NM_000075	CDK4	cyclin-dependent kinase 4	1.49	0.75	0.0429	regulation of cell cycle	0.0001
NM_001975	ENO2	enolase 2, (gamma, neuronal)	1.58	0.71	0.0432	energy derivation by oxidation of organic compounds	0.5979
AF249898		unknown	1.53	0.69	0.0432		
AK023787	FLJ13725	hypothetical protein FLJ13725	1.60	0.69	0.0432		
M38449	TGFB1	transforming growth factor, beta 1 (Camurati-Engelmann disease)	1.49	0.68	0.0432	regulation of cell cycle	0.0001
AF026816	ITPA	inosine triphosphatase (nucleoside triphosphate pyrophosphatase)	1.48	0.60	0.0454	nucleotide metabolism	0.6125
NM_002547	OPHN1	oligophrenin 1	1.51	0.54	0.0469	cell communication	0.0279
AK022226		unknown	1.81	0.45	0.0491		
NM_002292	LAMB2	laminin, beta 2 (laminin S)	1.58	0.41	0.0507	cell adhesion	0.3235
AL049987		unknown	1.42	0.39	0.0514		
AF086147		unknown	1.39	0.36	0.0531		
NM_003714	STC2	stanniocalcin 2	1.55	0.35	0.0531	response to external stimulus	0.0075
NM_002960	S100A3	S100 calcium binding protein A3	1.47	0.35	0.0535	pathogenesis/invasive growth	1.0000
NM_012266	DNAJB5	DnaJ (Hsp40) homolog, subfamily B, member 5	1.62	0.31	0.0535	response to stress	0.0356
NM_017606	DKFZp434K1210	hypothetical protein DKFZp434K1210	1.46	0.30	0.0535		
NM_005238	ETS1	v-ets erythroblastosis virus E26 oncogene homolog 1 (avian)	1.58	0.23	0.0557	negative regulation of cell proliferation	0.0023
NM_002192	INHBA	inhibin, beta A (activin A, activin AB alpha polypeptide)	1.56	0.20	0.0569	regulation of cell cycle	0.0001
NM_014600	EHD3	EH-domain containing 3	1.47	0.10	0.0608	cell growth and/or maintenance	0.2968
AB041269	LOC160313	keratin 19 pseudogene	1.44	0.09	0.0613	cell growth and/or maintenance	0.2968

**Table 7:** Two-sample comparison B-statistic analysis: transcripts significantly up-regulated in 25 μM transplatin treatments (relative to both DMF (control) and 1 μM cisplatin treatments) Transcripts are ranked in order of evidence for differential expression according to one-sample comparison B-statistics and corresponding P-values.

Accession #	Gene symbol	Full gene name	Fold-change	B-statistic	P-value	Representative gene category	EASE score
D17232		unknown	1.98	2.363	0.0196		
S81893		unknown	1.83	2.273	0.0206		
NM_003330	TXNRD1	thioredoxin reductase 1	1.78	2.186	0.0208	heavy metal sensitivity/resistance	0.0115
AK022848		unknown	1.59	1.708	0.0275		
NM_005953	MT2A	metallothionein 2A	1.61	1.147	0.0353	heavy metal sensitivity/resistance	0.0115
NM_002172	IFNA14	interferon, alpha 14	1.57	1.027	0.0370	receptor binding	0.1716
NM_003535	HIST1H3J	histone 1, H3j	1.71	0.725	0.0429		
NM_020313	LOC57019	hypothetical protein LOC57019	1.44	0.575	0.0460	catalytic activity	0.7398
NM_020121	UGCGL2	UDP-glucose ceramide glucosyltransferase-like 2	1.59	0.503	0.0486	catalytic activity	0.7398
AK025004		unknown	1.46	0.173	0.0581		
AK025447	ZNF336	zinc finger protein 336	1.43	0.125	0.0608	nucleobase\, nucleoside\, nucleotide and nucleic acid metabolism	0.1131
AL080110	PAQR3	progestin and adipoQ receptor family member III	1.37	0.081	0.0613	integral to membrane	0.7284

**Table 8:** Two-sample comparison B-statistic analysis: transcripts significantly down-regulated in 1 μM cisplatin treatments (relative to both DMF (control) and 25 μM transplatin treatments) Transcripts are ranked in order of evidence for differential expression according to one-sample comparison B-statistics and corresponding P-values.

Accession #	Gene symbol	Full gene name	Fold-change	B-statistic	P-value	Representative gene category	EASE score
NM_015885	PCF11	pre-mRNA cleavage complex II protein Pcf11	1.94	5.319	0.0033	mRNA binding	0.0286
NM_003234	TFRC	transferrin receptor (p90, CD71)	1.95	5.187	0.0033	transition metal ion homeostasis	0.0481
AF085846		unknown	1.83	4.802	0.0044		
NM_005066	SFPQ	splicing factor proline/glutamine rich (polypyrimidine tract binding protein associated)	1.86	4.310	0.0056	mRNA binding	0.0286
NM_002450	MT1L	metallothionein –1L	2.08	4.143	0.0058		
NM_001945	DTR	diphtheria toxin receptor (heparin-binding epidermal growth factor-like growth factor)	1.68	3.813	0.0075	positive regulation of cell proliferation	0.0394
U62823		unknown	1.79	2.616	0.0170		
NM_004093	EFNB2	ephrin-B2	2.06	2.245	0.0196	development	0.1884
NM_006910	RBBP6	retinoblastoma binding protein 6	1.81	2.085	0.0214	cell proliferation	0.3191
AK027074	KIAA1702	KIAA1702 protein	1.71	2.002	0.0218	phosphoenolpyruvate-dependent sugar phosphotransferase system	0.0425
NM_016109	ANGPTL4	angiopoietin-like 4	1.80	1.561	0.0290		
AK027174		unknown	1.97	1.362	0.0331		
AL133111		unknown	1.57	1.181	0.0353		
AB046797	ZSWIM6	zinc finger, SWIM domain containing 6	1.54	1.149	0.0353	regulation of protein activity\, epigenetic	0.0370
X73478	PPP2R4	protein phosphatase 2A, regulatory subunit B′ (PR 53)	1.62	1.104	0.0353	metabolism	0.4686
AK022171		unknown	2.36	0.947	0.0382		
NM_005667	RNF103	ring finger protein 103	1.46	0.863	0.0395	nucleic acid binding	0.0727
AK025835		unknown	1.44	0.728	0.0429		
AK023939		unknown	2.25	0.592	0.0468		

**Table 9:** Two-sample comparison B-statistic analysis: transcripts abundant in 25 μM transplatin treatments (relative to 1 μM cisplatin treatments) for which a one-sample origin of differential expression could not be determined.

Accession #	Gene symbol	Full gene name	FOLD-change	B-statistic	P-value	Representative gene category	EASE score
NM_003590	CUL3	cullin 3	1.59	3.196	0.0114	positive regulation of cell proliferation	0.0394
Z36838		unknown	1.69	2.274	0.0206		
AK024244	LOC284723	hypothetical protein LOC284723	1.92	2.201	0.0208		
AL133074	TP53INP1	tumor protein p53 inducible nuclear protein 1	1.64	2.065	0.0214		
D28450	H2AFZ	H2A histone family, member Z	1.46	1.882	0.0240	nucleobase\, nucleoside\, nucleotide and nucleic acid metabolism	0.1131
AK002085	LOC144438	hypothetical protein LOC144438	1.64	1.788	0.0257		
NM_006620	HBS1L	HBS1-like (S. cerevisiae)	1.55	1.623	0.0290	protein biosynthesis	0.7910
AK025119		unknown	1.45	1.549	0.0290		
AL110177		unknown	1.48	1.515	0.0292		
NM_017761	PNRC2	proline-rich nuclear receptor coactivator 2	1.55	1.510	0.0292	receptor activity	0.7020
AK026946	ARL6IP2	ADP-ribosylation-like factor 6 interacting protein 2	1.55	1.300	0.0338	response to external stimulus	0.8586
NM_004219	PTTG1	pituitary tumor-transforming 1	1.40	1.077	0.0360	transcription\ DNA-dependent	0.1145
AK025697	LOC200933	hypothetical protein LOC200933	1.53	1.068	0.0369		
NM_018514		unknown	1.62	1.005	0.0377		
NM_003814	ADAM20	a disintegrin and metalloproteinase domain 20	1.51	0.969	0.0381	sexual reproduction	0.4054
NM_014872	ZBTB5	zinc finger and BTB domain containing 5	1.51	0.800	0.0419	transcription\ DNA-dependent	0.1145
NM_004894	C14orf2	chromosome 14 open reading frame 2	1.44	0.726	0.0429		
NM_006460	HIS1	HMBA-inducible	1.61	0.644	0.0440	regulation of protein activity\ epigenetic	0.0370
NM_006385	ZNF211	zinc finger protein 211	1.52	0.642	0.0440	transcription\ DNA-dependent	0.1145
U56725		unknown	1.48	0.597	0.0460		
AK023371		unknown	1.78	0.588	0.0468		
NM_002858	ABCD3	ATP-binding cassette, sub-family D (ALD), member 3	1.43	0.346	0.0531	cell growth and/or maintenance	0.3678
NM_005461	MAFB	v-maf musculoaponeurotic fibrosarcoma oncogene homolog B(avian)	1.65	0.336	0.0535	transcription\ DNA-dependent	0.1145
NM_018138	FLJ10560	hypothetical protein FLJ10560	1.41	0.292	0.0535	carbohydrate transport	0.1018
NM_004730	ETF1	eukaryotic translation termination factor 1	1.46	0.261	0.0548	protein biosynthesis	0.7910
AK021725		unknown	1.70	0.185	0.0608		
NM_003749	IRS2	insulin receptor substrate 2	1.67	0.116	0.0608	positive regulation of cell proliferation	0.0394
AF070617		unknown	1.52	0.083	0.0613		
NM_006425	SLU7	step II splicing factor SLU7	1.44	0.057	0.0626	mRNA processing	0.0548
AK025020	AP1G1	adaptor-related protein complex1, gamma 1 subunit	1.46	0.053	0.0622	endocytosis	0.2677
AL133611		unknown	1.64	0.052	0.0622		
NM_017645	FAM29A	family with sequence similarity 29, member A	1.58	0.049	0.0622		
NM_004865	TBPL1	TBP-like 1	1.44	0.036	0.0635	transcription\ DNA-dependent	0.1145

**Table 10:** Two-sample comparison B-statistic analysis: The first 30 most significantly over-represented gene categories among transcripts more abundant in 1 μM cisplatin treatments (relative to 25 μM transplatin treatments) The number of transcripts in each gene category (List Hits) is also presented as a percentage of all the transcripts belonging to that category that were actually present on the microarray (Population Hits), and as a percentage of the 105 differentially expressed transcripts determined for that comparison (% Significant Transcripts). Categories are ranked in order of evidence for their over-representation among significant transcripts (EASE score).

Rank	GO system	Gene category	List hits	Population hits	% Population hits	% Significant transcripts (Total = 105)	EASE score
1	Biological Process	regulation of cell cycle	12	362	3	11	5.7 × 10^−5^
2	Biological Process	regulation of cellular process	9	311	3	9	0.0018
3	Biological Process	regulation of cell proliferation	8	243	3	8	0.0019
4	Biological Process	response to pest/pathogen/parasite	10	393	3	10	0.0021
5	Biological Process	negative regulation of cell proliferation	6	127	5	6	0.0023
6	Biological Process	cell cycle	13	653	2	12	0.0025
7	Biological Process	immune response	12	598	2	11	0.0038
8	Cellular Component	extracellular	17	1134	1	16	0.0041
9	Biological Process	cellular process	53	5588	1	50	0.0065
10	Biological Process	response to wounding	7	231	3	7	0.0066
11	Biological Process	response to external stimulus	17	1145	1	16	0.0075
12	Biological Process	defense response	12	657	2	11	0.0077
13	Biological Process	cell proliferation	15	988	2	14	0.0112
14	Cellular Component	extracellular matrix	7	288	2	7	0.0146
15	Biological Process	response to biotic stimulus	12	720	2	11	0.0147
16	Biological Process	cell communication	29	2744	1	28	0.0279
17	Biological Process	regulation of mitotic cell cycle	2	4	50	2	0.0288
18	Biological Process	inflammatory response	5	160	3	5	0.0291
19	Biological Process	signal transduction	24	2167	1	23	0.0325
20	Biological Process	innate immune response	5	166	3	5	0.0327
21	Molecular Function	cytokine activity	5	180	3	5	0.0329
22	Biological Process	cell-cell signaling	9	515	2	9	0.0332
23	Biological Process	response to stress	11	721	2	10	0.0356
24	Molecular Function	receptor binding	8	472	2	8	0.0388
25	Biological Process	morphogenesis	13	956	1	12	0.0427
26	Molecular Function	transforming growth factor-beta receptor binding	2	7	29	2	0.0462
27	Biological Process	cell surface receptor linked signal transduction	13	968	1	12	0.0463
28	Biological Process	cell cycle arrest	3	50	6	3	0.0512
29	Molecular Function	zinc ion binding	6	324	2	6	0.0667
30	Biological Process	cell motility	6	302	2	6	0.0688

**Table 11:** Two-sample comparison B-statistic analysis: The first 30 most significantly over-represented gene categories among transcripts more abundant in 25 μM transplatin treatments (relative to 1 μM cisplatin treatments) The number of transcripts in each gene category (List Hits) is also presented as a percentage of all the transcripts belonging to that category that were actually present on the microarray (Population Hits), and as a percentage of the 64 differentially expressed transcripts determined for that comparison (% Significant Transcripts). Categories are ranked in order of evidence for their over-representation among significant transcripts (EASE score).

Rank	GO system	Gene category	List hits	Population hits	% Population hits	% Significant transcripts (Total = 64)	EASE score
1	Biological Process	heavy metal sensitivity/resistance	2	4	50.0	3	0.0115
2	Cellular Component	intracellular	27	6576	0.4	42	0.0181
3	Molecular Function	mRNA binding	3	87	3.4	5	0.0286
4	Biological Process	regulation of protein activity\, epigenetic	2	13	15.4	3	0.0370
5	Biological Process	positive regulation of cell proliferation	3	109	2.8	5	0.0394
6	Biological Process	phosphoenolpyruvate-dependent sugar phosphotransferase system	2	15	13.3	3	0.0425
7	Biological Process	transition metal ion homeostasis	2	17	11.8	3	0.0481
8	Biological Process	mRNA processing	3	131	2.3	5	0.0548
9	Biological Process	mRNA metabolism	3	146	2.1	5	0.0663
10	Molecular Function	nucleic acid binding	13	2586	0.5	20	0.0727
11	Cellular Component	nucleus	13	2675	0.5	20	0.0882
12	Biological Process	carbohydrate transport	2	37	5.4	3	0.1018
13	Molecular Function	binding	24	6252	0.4	38	0.1078
14	Biological Process	nucleobase\, nucleoside\, nucleotide and nucleic acid metabolism	12	2620	0.5	19	0.1131
15	Biological Process	transcription\, DNA-dependent	9	1730	0.5	14	0.1145
16	Biological Process	di-\, tri-valent inorganic cation homeostasis	2	43	4.7	3	0.1173
17	Molecular Function	antioxidant activity	2	41	4.9	3	0.1176
18	Molecular Function	DNA binding	10	1944	0.5	16	0.1219
19	Molecular Function	sugar porter activity	2	44	4.5	3	0.1256
20	Biological Process	metal ion homeostasis	2	48	4.2	3	0.1301
21	Molecular Function	carbohydrate transporter activity	2	50	4.0	3	0.1415
22	Molecular Function	RNA binding	4	437	0.9	6	0.1460
23	Biological Process	regulation of cell proliferation	3	243	1.2	5	0.1554
24	Biological Process	cation homeostasis	2	63	3.2	3	0.1673
25	Molecular Function	receptor binding	4	472	0.8	6	0.1716
26	Molecular Function	pre-mRNA splicing factor activity	2	63	3.2	3	0.1750
27	Biological Process	regulation of gene expression\, epigenetic	2	69	2.9	3	0.1817
28	Biological Process	cell ion homeostasis	2	70	2.9	3	0.1841
29	Biological Process	development	8	1646	0.5	13	0.1884
30	Biological Process	regulation of transcription\, DNA-dependent	8	1660	0.5	13	0.1942

## Abbreviations

bp: base pair
cisplatin: *cis*-diamminedichloroplatinum(II)
transplatin: *trans*-diamminedichloroplatinum(II)
